# Situational analysis of teaching and learning of medicine and nursing students at Makerere University College of Health Sciences

**DOI:** 10.1186/1472-698X-11-S1-S3

**Published:** 2011-03-09

**Authors:** Sarah Kiguli, Rhona Baingana, Ligia Paina, David Mafigiri, Sara Groves, Godfrey Katende, Elsie Kiguli-Malwadde, Juliet Kiguli, Moses Galukande, Mayega Roy, Robert Bollinger, George Pariyo

**Affiliations:** 1 Makerere University College of Health Sciences, Kampala, Uganda; 2 Faculty of Science, Makerere University, Kampala, Uganda; 3 School of Public Health, Johns Hopkins University, Baltimore, MD 21205, USA; 4 Faculty of Social Sciences, Makerere University, Kampala, Uganda; 5 School of Nursing, Johns Hopkins University, Baltimore, MD 21205, USA; 6 School of Medicine, Johns Hopkins University, Baltimore, MD 21205, USA; 7 HQ/HWA Global Health Workforce Alliance, World Health Organization, Geneva, Switzerland

## Abstract

**Background:**

Makerere University College of Health Sciences (MakCHS) in Uganda is undergoing a major reform to become a more influential force in society. It is important that its medicine and nursing graduates are equipped to best address the priority health needs of the Ugandan population, as outlined in the government’s Health Sector Strategic Plan (HSSP). The assessment identifies critical gaps in the core competencies of the MakCHS medicine and nursing and ways to overcome them in order to achieve HSSP goals.

**Methods:**

Documents from the Uganda Ministry of Health were reviewed, and medicine and nursing curricula were analyzed. Nineteen key informant interviews (KII) and seven focus group discussions (FGD) with stakeholders were conducted. The data were manually analyzed for emerging themes and sub-themes. The study team subsequently used the checklists to create matrices summarizing the findings from the KIIs, FGDs, and curricula analysis. Validation of findings was done by triangulating information from the different data collection methods.

**Results:**

The core competencies that medicine and nursing students are expected to achieve by the end of their education were outlined for both programs. The curricula are in the process of reform towards competency-based education, and on the surface, are well aligned with the strategic needs of the country. But implementation is inadequate, and can be changed:

• Learning objectives need to be more applicable to achieving competencies.

• Learning experiences need to be more relevant for competencies and setting in which students will work after graduation (i.e. not just clinical care in a tertiary care facility).

• Student evaluation needs to be better designed for assessing these competencies.

**Conclusion:**

MakCHS has made a significant attempt to produce relevant, competent nursing and medicine graduates to meet the community needs. Ways to make them more effective though deliberate efforts to apply a competency-based education are possible.

## Background

Educational institutions are key to training health professionals who can meet people’s needs, empower communities, and enhance human wellbeing [1]. Therefore, reforming the vision and implementing state-of-the-art teaching methods for medicine and nursing education programs are key to building a competent health workforce, a particular concern in low and middle income countries. During the past decade most medical and nursing curricula in high income countries have undergone a paradigm shift from content and process-based education to outcome or competency-based models of education, which focus on linking the educational process directly to workforce needs and expectations [2-4].

Sub-Saharan African (SSA) universities have also taken initiative for curriculum reform, albeit progressing at a slower pace and on a smaller scale. Similar to the early phases of curricula reform in high-income countries, the shift in Sub-Saharan Africa has been away from traditional learning methods towards the development of process-based education. Universities have been implementing process-based education through Problem-Based Learning and Community-Based Education Models. In health professions education, these models, which have been documented in the published literature in Ethiopia, South Africa, Uganda, and Zimbabwe, are characterized by student-focused small group learning, community-based experience, and improved assessment methods [5-12].

An even smaller number of universities, Makerere being among them, are in the early phases of moving towards outcome-based or competency-based curricula, which focus on preparing students to enter the workforce armed not only with excellent clinical skills, but also with the ability to think critically and to display good interpersonal skills. These early phases manifest themselves with adaptations of problem-based learning curricula to increasingly focus on student outcomes, early clinical exposure, and integrating competencies throughout the entire undergraduate coursework [7,10,13,14]. The emerging nature of this movement provides an immediate opportunity for medical and nursing education experts in Sub-Saharan African universities to document and evaluate the extent to which current programs define competencies and to support a further shift towards outcome-based education.

Makerere University, one of the oldest universities in Africa, was established in 1922. Makerere University College of Health Sciences (MakCHS), established in December 2007, evolved from the University’s Faculty of Medicine and School of Public Health. The largest medical training institution in Uganda, MakCHS is currently comprised of the Schools of Medicine, Public Health, Biomedical Sciences, and Health Sciences. This study was conducted at this time because of the current re-organization at Makerere University with the establishment of a Makerere College of Health Sciences (MakCHS), which is being undertaken to have a more effective impact on society in Uganda and internationally [15]. The University has long been a major contributor to the production of the health workforce in the Ugandan health system, and the new vision for MakCHS is to be a transformative institution and leader in health professions education, research and service, with a broader impact on health and society in Uganda and beyond [16].

The authors present findings from an assessment of the medicine and nursing programs which was conducted to determine the degree to which the MakCHS prepares graduates to support improvements in key health outcomes in Uganda. More specifically, the article first describes the organization of and recent developments in the medicine and nursing curricula. Then, it discusses the gap between the core competencies currently included in the medicine and nursing curricula and those necessary to achieve the goals of the Health Sector Strategic Plan (HSSP) in the priority areas of maternal and child health, mental health, environmental health and infectious diseases, such as malaria, HIV/AIDS, and tuberculosis [17]. Lastly, the article presents a set of key opportunities to leverage during the upcoming medical and nursing curricula revisions. As curriculum reform in Sub-Saharan Africa is a relatively new undertaking, the findings and reflections presented in this paper will inform similar processes in other Sub-Saharan African Universities which are challenged with developing a competent medical and nursing health workforce.

## Methods

The study team was comprised of faculty from all MakCHS schools, as well as social scientists from Makerere University. Colleagues from Johns Hopkins University also worked closely with the study team, particularly on initial design and proposal preparation. The descriptive, cross sectional needs assessment was conducted between November 2009 and February 2010 using participatory qualitative methodology.

### Data collection

Data collection involved document review, key informant interviews (KII), focus group discussions (FGD), and curricula analysis using guides developed specifically for this study.

### Document review

A thorough document review of the latest HSSP [17] and related documents, such as the Ugandan National Health Policy [18], Ugandan Health Sector Strategic Plan II [17], Ugandan Child Survival Strategy [19], Road Map to Maternal Health [20], as well as Ministry of Health (MoH) Reports on Malaria [21], Tuberculosis, and HIV/AIDS [22], was conducted in order to identify the HSSP goals which guide the competencies needed by health workers. The document review also informed the development of FGD and KII guides and the curriculum analysis tools, all of which contributed to the comparison between the competencies currently included in the curricula and those which are needed to accomplish HSSP goals and the implementation of the Uganda Minimum Health Care Package.

### Key Informant Interviews and Focus Group Discussions

Study participants included key external and internal MakCHS stakeholders purposively selected by the study team. The external stakeholders included important health sector actors, including policy makers, program managers, professional councils, health consumer organizations, employers of MakCHS graduates, alumni working as interns at Mulago Hospital, and higher education standards regulators. Internal stakeholders included MakCHS leadership, faculty, students and alumni of the undergraduate programs currently in post-graduate programs.

The study team conducted a total of nineteen KIIs. The KIIs with external stakeholders included officials from the following Departments of the Ugandan MoH: Community Health, Planning, Child Health, and Curative and Reproductive Health. These officials were selected based on their position as leaders in the Ugandan health sector, their direct involvement in and oversight of activities aimed at realizing the HSSP goals, and their rich understanding of the health workforce requirements of the Ugandan health system. Key informant interviews were also conducted with the Registrars of the Nursing and Medical and Dental Professional Councils, representatives of the Uganda Health Care Consumer Organization and of the National Council for Higher Education. The District Health Officers of Mukono, Soroti, Iganga, Rakai, and Mpigi districts, where MakCHS students participate in field learning experiences, were selected to represent health professionals outside MakCHS who are both directly and indirectly involved in student training as well as in supervision of the graduates of MakCHS working in the district health facilities. In addition, the study team conducted KIIs with representatives of the Health Service Commission and the World Health Organization.

The KIIs with internal stakeholders included: the Principal of MakCHS, Deans from the Schools of Medicine, Health Sciences, and Public Health, and Heads of the Departments of Nursing, Psychiatry, Environmental Health, Pediatrics & Child Health, Internal Medicine, and Obstetrics & Gynecology.

Seven FGDs were conducted with MakCHS students, alumni, and faculty. Of these, two were conducted with students, two with alumni, and three with faculty of MakCHS. Each FGD was comprised of five to eight participants. Faculty participants included a cross-section of teaching staff from the MakCHS’s Department of Nursing, School of Public Health and School of Medicine. The study team selected them from departments directly linked to HSSP priorities (e.g. Maternal and Child Health, Mental Health, Infectious Disease, TB/HIV/AIDS, Malaria, and Preventive Medicine). Among the student body, the team approached student leaders to help identify student FGD participants. Alumni were selected from the pool of current residents and interns at Mulago Hospital with the help of a MakCHS alumnus that the study team was familiar with. The study team approached heads of Departments for permission to inform their staff about the study and invite them to participate in the FGDs. The student leaders of medical and nursing students were also informed about the study and requested to call the study participants for the students FGDs. Respondents were invited according to their availability. None of the KII respondents participated in the FGDs. Similarly, the team took deliberate steps to ensure that FGD participants, such as alumni, did not participate in KIIs.

The KII and FGD guides designed specifically for this study sought the respondents’ views about the current status, gaps, and future needs relative to HSSP goals and with respect to the medical and nursing graduates’ competencies and learning experiences. At the beginning of each FGD or KII, the team explained the study purpose to respondents and obtained verbal consent, including consent for audio recording. Written informed consent was obtained from student and alumni FGD participants. The FGDs were conducted by teams comprised of both clinical and nonclinical researchers with experience in qualitative research methods. Each FGD was conducted by a moderator and a note taker, and were audio recorded. The notes and audio recordings were transcribed immediately after the sessions. Both FGDs and KIIs were conducted in English, the official language of Uganda. Study participants did not receive any monetary compensation.

### Curriculum analysis

The study team analyzed the current medical and nursing curricula in order to identify the stated core competencies and program outcome objectives. Individual courses were then examined to determine whether they integrated the identified core competencies and, if so, whether faculty included methods to establish the degree to which students achieved them upon graduation. The analysis also looked at the content of the courses to examine if they addressed the priority areas of the HSSP, as well as if the learning of all competencies was achieved. The curriculum analysis was conducted with the aid of checklists developed by team members with expertise in curricula review and qualitative research. The checklist items consisted mainly of items requiring respondents to mark the competencies currently outlined in the curriculum, the areas of HSSP already covered in the curricula content, and whether competencies and related assessment methods were integrated in the courses’ learning objectives.

### Data analysis

The study team jointly reviewed and manually analyzed the transcripts for emerging themes and sub-themes. The study team organized the themes in the following categories: gaps and future needs in number and types of students required to meet the HSSP goals, gaps and future needs of the curricula, and competencies. The study team subsequently used the checklists to create matrices summarizing the findings from the KIIs, FGDs, and curricula analysis.

The team validated their findings by triangulating the information obtained from a wide range of stakeholders who participated in the study as key informants and as focus group discussants. Furthermore, preliminary findings were presented to stakeholders at a meeting in February 2010, which provided an opportunity for feedback, assessment of credibility, and confirmability. In order to assess the degree of dependability, the team ensured that the methods of this study are transparent and replicable in other SSA University settings and that they summarized the major limitations to this study. While the findings of this study will likely not be directly transferable to other contexts, their usefulness lies in documenting curricula reform at MakCHS for future use within the University and helping other institutions better frame their own curricula assessments.

The findings from all the data sources (FGD, KII, and Document Review) were examined by the team jointly and the Recommended Core Competencies were identified and mutually agreed upon (see Table 1). Competencies Recommended Core Competencies

**Table 1:** Comparison between essential core competencies recommended by KI and FGDs and current curricula

Core competencies recommended in FGDs and KII	Medicine Curriculum (Source: 2003 curricula revision)	Nursing Curriculum (Source: 2003 curricula revision)
***Good alignment between stated core competencies and FGD and KII recommendations, yet poor systematic integration into course objectives and learning activities.***		

Knowledge (discipline, medico-legal, population, health and health systems)	present	present
Discipline specific clinical skills for patient management (history taking, assessment of patient, performance of procedures, and formulation of a management plan)	present	present
Professional development skills (lifelong learning)	present	present
Education	dispersed	present, required course

***Highlighted by FGD and KII as areas not adequately addressed in the implementation of nursing and medicine curricula***		

Communication and interpersonal skills	present	present
Professional behavior	present	present
Health promotion and disease prevention	present	present
Community health skills	present	present

***Highlighted by FGD and KII as areas not adequately addressed in the implementation of nursing and medicine curricula. Present at varying levels in the two curricula.***		

Research	dispersed	present, required course and research study
Management and Leadership	dispersed	present, required course

***Core competencies recommended by FGD and KII but not in the core curricula. This highlights the gaps between competencies and curricula necessary to meet Ugandan health goals***		

Systems-based practice	absent	absent
Continuous improvement of services	absent	absent
Information Technology skills	absent	absent

### Ethical approval

The study was approved by the Institutional Review Boards of Makerere University School of Public Health and Johns Hopkins University School of Public Health.

## Results

### Recent developments in the current medical and nursing curricula

The medical and nursing curricula at MakCHS are each organized in three phases. Phase I is interdisciplinary and conducted jointly for the first two years of the medical and nursing programs. It emphasizes the pre-clinical sciences and the acquisition of knowledge. Phase II and III gradually build upon this initial knowledge foundation with clinical skills training as well as rotations, and are organized around organ systems. Teaching and learning of the biomedical sciences (anatomy, physiology, and biochemistry) is integrated. There is some minimal clinical integration in this early phase through basic clinical exposures, case studies and some basic clinical skills training. During Phase I, students also get introduced to behavioral sciences, epidemiology and biostatistics. For the medical program, Phase II occurs in the third year and Phase III in the fourth and fifth. Phase II addresses abnormal body structure and function, and emphasizes pathophysiology of systemic disorders, as well as pharmacology. Phase III of the medical program emphasizes both knowledge and clinical outcomes. This phase involves students rotating through different clinical disciplines. For the nursing program, Phase II and III occur during the third and fourth years of the program.

The curricula are delivered mainly through student-centered learning approaches including problem-based learning, early clinical contact with patients, clinical experiences with patients during clinical clerkships, and community-based education and service (COBES). COBES starts during Phase I of both programs, and continues through Phase II of the nursing program and Phase III of the medicine program.

### Current core competencies

The curriculum analysis revealed that that the core competencies that medical and nursing students are expected to achieve by the end of the training were clearly outlined for both programs (Table 1). While the core competencies outlined by the two curricula were similar, nursing puts more emphasis on research skills, management, education and professional development competencies than the medicine curricula. These stated core competencies as well as the clinical content were, in general, well aligned with those identified by KI and FGDs as necessary to meet HSSP targets in maternal and child health, mental health, environmental health, and infectious diseases [16].

However, the curriculum analysis also revealed that the core competencies were not systematically integrated into all the course objectives and learning activities. Furthermore, the course objectives of Phase I and II were mainly designed in the cognitive domain and generally reflected lower cognitive levels of knowledge and comprehension (i.e. “to describe” and “to explain” were the most common verbs used in the objectives for the biomedical sciences, in contrast to those which would reflect knowledge acquisition at higher domain levels, such as “to apply” and “to analyze”). Even in the instances where the language was more explicit, the metrics and processes by which faculty evaluated student competencies were not outlined. Furthermore, the link among core competencies, coursework, and expected student outcomes during their progression through the program, was difficult to establish.

The observations recorded from the KIIs and FGDs provided further insights into the strengths and weaknesses of curricula implementation. A district health officer explained that *“the current curriculum for Medical Officers mostly covers clinical aspects*, *specifically patient management. This aspect of the training is well done. Even the internship that medical officers go through before they are registered mainly covers clinical skills.”* However, the concerns raised by study participants indicated that the current teaching and learning strategies did not adequately address some of the stated core competencies, for example, leadership and management, interpersonal communication, professionalism, and primary care.

As an MoH official explained, *“Management is one of the things that we lack in this country and really to be able to use available data but that is all management. Management is a very key issue in Africa managing the available resources and being able to produce results in the area key issues. How do you manage available resources? How do you manage the money available to you?”* On a related note, the district officials and international agencies interviewed noted that all health professionals, not just doctors and nurses, needed some leadership skills to manage and create the change necessary to meet the HSSP targets. They also emphasized the need for team building to facilitate cooperation both within and among the different health system operational levels. In general, most participants agreed, as a MoH director stated, that “*The modern doctor also needs good management and leadership skills.”*

Respondents also expressed repeated concerns that the health professionals did not have the necessary interpersonal skills to communicate well with staff, community and patients. This finding resonates throughout the curriculum analysis, which also found that most of the course objectives targeted cognitive and psychomotor outcomes, with less emphasis on affective outcomes and other competencies. A representative of the Health Consumers Organization affirmed: *“There is need to train people in humanistic skills which will help them to learn how to interact with people whether patients or not patients*, *respect*, *treat people with empathy*, *have a listening ear*, *listen and understand people*, *learn to deal with human beings as human beings and understand them as human beings.”*

Lack of professionalism in practice, including ethics, integrity, and physical presence in the health facilities, was another gap identified in the core competencies of the medicine and nursing graduates. Respondents said that ethical conduct of health workers was a key issue and argued that MakCHS students should be sensitized on human and patient rights.

Other key respondents raised the issue of faculty and student integrity as well as their attitudes toward medical and nursing practice. These respondents emphasized the role of faculty in modeling good behavior and mentoring students. According to alumni who participated in FGDs, the issue of professionalism was compounded by the lack of role models within the faculty to model this competency. One alumnus posited: *“Now the ethics*, *well*, *it has to be in you*, *the integrity. So if the person who teaches it doesn’t behave that way then I also wouldn’t really have the guts to follow unless I have that personal*, *you know touch. I know what it means to have integrity*, *respect and all that. So you find that everyone is taught ethics but some of the seniors don’t even behave in the ethical way.”*

A final major gap in the current curricula that was emphasized throughout the findings was an insufficient emphasis on primary prevention, which includes both health promotions and disease prevention. As one of the district health officers stated, *“There is little emphasis on preventive and promotion of health service delivery. There is a lot of emphasis on curative*, *investigative*, *good clinical history and examination which are more expensive.”* In a separate KII, a MakCHS Dean agrees: *“Even another thing that we have also realized is needed for achieving the Health Sector Strategic Plan targets is actually people going out for preventive services. They tend to be ignored in the training. I think it is not focused on a lot. You find that even a clinical officer does not prioritize going out to focus on hygiene and sanitation so that he educates; he knows a lot*, *but he doesn’t see it as his role.”*

Whereas the MoH official emphasized the clinical skills for quality patient management was a very important core competency for MakCHS graduates, the district health officers as well as officials from the development partners put leadership and management as the main key competency health professionals had to possess in order meet the HSSP targets. Representatives of health consumers’ organization put the emphasis on the need for health professionals to empower communities to promote their health and to possess the competency of good communication skills. However, the importance professionalism, communication and good interpersonal skills as core competencies of graduates of MakCHS were highlighted by all the study participants.

### Alignment of current competencies with HSSP and implications for learning experiences

Although the curricula seem to be aligned with the HSSP on paper (see Table 1), respondents indicated that HSSP goals were not adequately integrated into the practical learning experiences of MakCHS students. A program manager with the WHO Uganda Country Office respondent explains: *“All modules should be wrapped up by presenting to the students the ‘program priorities’ and ‘global or regional targets’ and the priority interventions for achieving them. Apart from the Public Health courses*, *the input by resource persons from the WHO*, *UN Agencies and MoH in co-facilitating the training sessions is low. The College must look for other trainers from outside the regular teaching staff.”* At the same time, other respondents thought that the disconnect between the current competencies and those necessary to meet Uganda’s health targets should not only be explored from the school’s perspective, but also from the MoH’s. They believed that the MoH should also become more engaged in defining its health professionals’ education needs. One of the district health officers said that *“The current National Health Policy does not prescribe the balance in training that is needed to meet the HSSP targets; it does not prescribe specific areas that should be addressed by the training. It assumes that once a nurse or doctor is trained and they have the qualification*, *they should be able to run the health system. This is wrong. Who should train them in how to implement the HSSP service package?”*

### Guidance for the future

In addition to shedding light upon some of the critical gaps in the medical and nursing curricula at MakCHS, respondents offered several solutions for improving medical and nursing education at MakCHS and for increasing the alignment of the curricula goals and objectives to the needs of the Ugandan population. These solutions concentrated primarily around decentralization of training, strengthening partnerships with the MoH and other key stakeholders, and filling the highlighted gaps in the curricula.

Several respondents suggested that training should be decentralized to lower level health units in order to strengthen the critical linkages needed between the community and the health system and to better prepare students to deliver the Minimum Package of Health Services of cost effective interventions high impact interventions to serve the primary needs of the Ugandan population [16]. A faculty member who participated in FGDs suggested that MakCHS explore the *“use of resources surrounding us and changing the model; the students should be able to go to the City Council clinics* (*lower level health facilities*) *in order for them to learn better for example students in year one should not go to the wards of a tertiary hospital*, *but to the City Council clinics. Tutorials should be done at the health centers so they actually see patients with minor problems.”* One of the MakCHS Deans explained that: *“Mulago as the National Referral Hospital is sometimes too specialized for undergraduate training. College of Health Sciences could do better by affiliating and sending our students to regional hospitals where there are interns as well as Faith-based hospitals around Kampala to make learning interactive and the teachers would give supervisory support to these centers and offer continuous professional development. This would move a large part of teaching and learning to the community and increase the accountability of College of Health Sciences to the community.”*

Not only would increased training in the community strengthen the linkages between the future health workforce and the Ugandan population, but also improve the relevance of the students’ learning experiences to their needs.

Internal stakeholders also highlighted the need to strengthen curricula by involving the MoH and other stakeholders. One key informant from the MakCHS leadership recommended to *“work closely with MoH in planning the training program so that the curricula satisfy the need for the MoH. Work closely with MoH to define competencies once the MoH has defined the roles.”* Several other respondents also emphasized that all stakeholders, internal and external, should jointly develop training programs in order to optimize the set of competencies that graduates acquire and to ensure that they meet the needs of the Ugandan population.

When discussing opportunities to enhance the core competencies present in the curricula, addressing the gaps in leadership, management, communication and interpersonal skills, and professionalism were identified as high priorities for MakCHS in the next 10 years. In addition, respondents emphasized the need to strengthen students’ experiences within the community-based health care system. One of the district health officer interviewed emphasized the need *“ to train people in the areas where they are going to be posted like to have an attachment because I’ve noticed when we get people who have come during their training and get attached through rural districts they tend to stay.”*

Other respondents highlighted the need to enhance supervision of students and teamwork. For example, a MakCHS leader thought that *“supervision should be a factor too because it brings in mentoring. The team approach should be emphasized not only to learning but to solving problems so that as people move on*, *they move as teams but not as individuals.”* In addition to these issues, the need to emphasize training in ethics and human rights was also highly emphasized in discussion. According to a representative of the Health Consumer Organization, *“the providers should also know what the rights of patients are. And accountability and efficiency will come out if the users know what they need and how to demand for it and how to get it and how to hold providers and policy makers*, *it is not just the health workers they need to be able to demand from policy makers”.*

Respondents also highlighted the need of reviewing the existing curricula in order to accommodate the gaps in the competencies and learning experiences of the current programs. According to a MoH Department Head, “*curriculum reviews to improve*, *training should involve more community training*, *teachers to go in community with students which will be a spin off for continuous professional development.”*

## Discussion

Our findings indicate that the core competencies expected to be achieved at the end of MakCHS medicine and nursing programs are outlined by both curricula. Furthermore, as stated, the core competencies were generally well aligned with those identified by KII and FGDs as necessary to meet HSSP targets and to address community health. Additionally, the current medical and nursing programs were thought to have adequate clinical content to address the HSSP targets. The latter finding, in particular, is not surprising, as the two programs were developed with a goal of addressing the health needs of the community [12]. This process has involved a number of internal and external stakeholders, who identified the relevant competencies, as well as learning methodologies for the curricula [12]. It is therefore not surprising that the core competencies expected to be achieved by MakCHS nursing and medicine graduates are similar to those recommended in the Europe and North America [3,23]. In addition, there is overlap in the expected core competencies of nursing and medical students as has been observed by other studies [24].

However, the competency-based approach was lost in the implementation of most courses, or building blocks of the curricula, which emphasize mainly knowledge competency in Phase I and II of the programs, and knowledge and clinical skills competencies in Phase III. As a result, other competencies are not adequately addressed and graduates are likely to demonstrate these gaps in their professional practice. Developing competency-based programs at MakCHS, after defining appropriate levels of competence at each phase of learning, for medical and nursing students would address this gap. Furthermore, the competencies of professionalism, leadership and management need specific learning objectives, educational opportunities, mentoring, and appropriate evaluation.

Our study found that the organization of both medicine and nursing curricula is based on content, organized around organ systems. The organ-based reform which started in the 1950’s was a response to the growing amount of biomedical knowledge and disorganization inherent in a discipline-run curriculum [2,25]. It is important to note that MakCHS has made some attempt to take on such reforms in medical and nursing education.

However, there is no clear plan of assessment of students’ achievement of the desirable competencies. MakCHS should therefore develop a plan to match assessment methods with the competencies being learnt by the students as assessment drives students learning [26]. Performance-based assessment should be utilized for the difficult-to-assess competences such as professionalism, communication skills, team work that are not currently assessed.

The curricula also emphasize the process of learning. The current medical and nursing programs are implemented through state-of-the-art learning experiences, such as problem-based learning and community-based education and services (COBES). MakCHS has made strides in implementing the problem-based learning methodology that promotes student-centered learning, as well as critical thinking and problem solving skills [27,2]. Similarly, COBES was introduced to help students understand community health and primary care needs and to bring services closer to communities. Students have opportunities to experience other learning methods which include learning in hospital setting, that allow them to practice, and gain competency in clinical skills, and patient management. If well planned, these learning experiences can also be used to facilitate the students to learn the competencies that the stakeholders found to be lacking among current graduates. At MakCHS, there are opportunities for inter-professional education, which allows the medical and nursing students to understand each other’s training, roles, strengths and limitations and provides opportunities for team learning [2]. It is therefore important that these innovations are consolidated in order to produce medical and nursing graduates with desired competencies to address health needs of the Ugandan population.

Our study identified some gaps that need to be addressed during the current training of medical and nursing students at MakCHS. One gap lies in the way learning objectives of courses were written, emphasizing mainly knowledge outcome in Phase I and II and clinical skills for patient management in Phase III. In addition, the curriculum does not reflect the expected increases in the levels of learning with more years on the programs. Other areas that need to be improved include enhancing case-based learning and implementing interdisciplinary learning in Phase III of the curricula and creating clinical learning opportunities at primary or secondary level health facilities to enhance learning of primary and preventive care. Clinical teaching currently occurs at tertiary hospital which does not adequately prepare graduates for primary care. Lastly, problem-based learning is not adequately implemented in Phase III. Our study found that partnerships and collaborations with both internal and external stakeholders are important for MakCHS and other Sub-Saharan African health professions training institutions to introduce and sustain reforms [28]. Furthermore, these partnerships are crucial in order for health profession schools to achieve social accountability [29].

Our study had a number of limitations. At the time of data collection, the nursing graduates who were admitted in 2003 (when the new curriculum started) had been in the field (post internship) for only two years. The medical graduates had been in the field for only one year. Moreover, many of these graduates may not have had the chance to serve in district or rural health facilities. It is therefore possible that the comments made by the participants reflect the graduates of the old traditional curriculum. This needs assessment could not assess the real contribution of the current graduates who have had COBES and program-based learning experiences. Another limitation is that while the study was ongoing, a new draft HSSP was issued for comment. While the latest HSSP, currently in draft, has not been used for this study, the study team reviewed it briefly and concluded that there was sufficient overlap between both to use HSSP II. Lastly, faculty from MakCHS participated in the process of data collection, which could be a source of bias in the results.

## Conclusions

In conclusion, MakCHS has made considerable strides towards being socially accountable to the society through its efforts to produce medicine and nursing graduates with adequate competencies for meeting the priority health needs of the Ugandan population. It is critical that MakCHS continues to strengthen its efforts to develop and implement innovative medical and nursing education curricula. It is pertinent to seek partnership with all key stakeholders, particularly those with extensive experience in the process of developing and implementing relevant curricula, using innovative education methods.

## Competing interests

The authors declare that they have no competing interests.

## List of abbreviations used

MakCHS: Makerere University College of Health Sciences; HSSP: Health Sector Strategic Plan; KII: key informant interviews; FGD: focus group discussions; SSA: Sub-Saharan Africa; COBES: community-based education and service; MoH: Ministry of Health.

## Authors’ contributions

SK and RB participated in the conception and design of the study, participated in the data analysis, and writing of the manuscript. DM, GK, EK, MG, and JK participated in the conception and design of the study and in the review of the manuscript. LP and SG participated in the analysis and writing of the manuscript. RB and GP participated in the conception and design and review of the manuscript. All authors read and approved the final manuscript.
